# Editorial Comment: Acute prostatitis after prostate biopsy under ciprofloxacin prophylaxis with or without ornidazole and pre-biopsy enema: analysis of 3.479 prostate biopsy cases

**DOI:** 10.1590/S1677-5538.IBJU.2019.0257.1

**Published:** 2020-01-13

**Authors:** Andre Luiz Lima Diniz

**Affiliations:** 1 Instituto Nacional do Câncer - INCA, Rio de Janeiro, RJ, Brasil, Brasil; 2 Hospital Federal da Lagoa, Rio de Janeiro, RJ, Brasil

With great relevance, the International Brazilian Journal of Urology brings to its readers important material that points us to the dreaded complication inherent in transrectal ultrasound-guided prostate biopsy (TRUS-Bx) and the role of antibiotic prophylaxis against prostatitis (1). In their results, the authors demonstrated that cleasing enema did not change the outcome when comparing the groups. It has been reported a significantly lower rate of complications by administering glycerin or saline enema 1h before TRUS-Bx (2) although a randomized study has shown that the use of iodine-povidine solution would not have the potential to reduce the risk of prostatitis with statistical significance (3).

It should be agreed that even if it does not make the procedure aseptic, local hygiene is part of good practices, with low cost and adequate tolerability. In our clinical routine, we provide the patient, at the time of the biopsy indication, a glycerin suppository to be introduced into the rectum the night before the procedure. Among the advantages of this tactic, the patient can arrive with the empty rectum the next morning, and still facilitate the introduction of the probe by additional lubrication performed also with transrectal 2% lidocaine jelly anestesia after patient's sedation.

The number of samples collected and the diameter of the needle was also the subject of concern on the correlation with infection after TRUS-Bx. Authors used the 18G needle and harvesting of 10 fragments under local anesthesia.

Although studies suggest sampling with 12 to 18 fragments (4), the latest UAE recommendations propose that at least eight systematic biopsies are recommended in prostates with a size of about 30 cc and ten to twelve core biopsies are recommended in larger prostates (5). Ultrasound-guided periprostatic block is recommended (6) and is not related with increase rates of prostatitis (7).

Although bacterial resistance to ciprofloxacin is well reported (8–10), this drug still represents an affordable and effective option in the context of prostate biopsy prophylaxis against infections. As in our daily practice at the National Cancer Institute's Prostate Cancer Diagnostic Center, group 1 used ciprofloxacin for a total of four days. Statistical analysis have shown that addition of the second drug (ornidazole) did not significantly reduce the rate of prostatitis. In a public health scenario, this information is relevant as it allows us to save resources that could be used for other inputs.

Also regarding the relevant results, the authors report us the increased incidence of acute prostatitis in those individuals who underwent multiple TRUS-Bx.

This may be the outmost data brought in this paper. Realize how current this information is and why it should be highlighted: understanding that we are increasingly diagnosing individuals in the early stages of the disease and that management through active surveillance includes serial biopsies; when recommending subsequent procedures, the practitioner should be extremely alert to signs of complications and the patient should be aware of this increased risk (11).

Multiparametric MRI (mpMRI) is part of the active surveillance protocol for prostate cancer and has the potential to improve accuracy through ultrasound-guided target biopsies e and reduce overall complications (8, 12). Some authors evaluated it's use even in virgin individuals from previous biopsies (13), but neither the European (EAU/ ESTRO/SIOG/ESUR) nor the American (NCCN) guidelines endorse wholeheartedly mpMRI in biopsy-naïve men (14, 15). Using this technology to reduce the number of samples collected could virtually reduce complications associated to the procedure.

The authors acknowledged some limitations of their study, which indicates an important commitment of that team regarding the scientific communication. In this sense, I propose to the reader to remember that local realities sometimes determine specific behaviors not always aligned with international guidelines. In developing countries, limited access to health technologies is a clear sign of the social disparity of these societies. There are cases in which patients come to the diagnostic procedure already in a degrading state of health, sometimes catheterized and with UTI; in other cases, after the biopsy has been performed and the prescription provided, the patient has no way to get the drugs. These are everyday situations and it is up to us to make the best medical art, *primum non nocere*.

It is wonderful that we have advanced the level of knowledge and that humanization in health has reached the core of the most mustached urologists; but our efforts will be worthless if health access policies are not improved.
